# Differential Regulation of miRNA Profiles of Human Cells Experimentally Infected by *Leishmania donovani* Isolated From Indian Visceral Leishmaniasis and Post-Kala-Azar Dermal Leishmaniasis

**DOI:** 10.3389/fmicb.2020.01716

**Published:** 2020-07-31

**Authors:** Ashish Kumar, Saravanan Vijaykumar, Manas Ranjan Dikhit, Kumar Abhishek, Rimi Mukherjee, Abhik Sen, Pradeep Das, Sushmita Das

**Affiliations:** ^1^Department of Biochemistry, Rajendra Memorial Research Institute of Medical Sciences, Patna, India; ^2^Department of Bioinformatics, Rajendra Memorial Research Institute of Medical Sciences, Patna, India; ^3^Department of Molecular Biology, Rajendra Memorial Research Institute of Medical Sciences, Patna, India; ^4^Department of Microbiology, All India Institute of Medical Sciences, Patna, India

**Keywords:** PKDL, miRNA, TGF-β, T cell, immune regulation

## Abstract

MicroRNAs are small ribonucleic acid that act as an important regulator of gene expression at the molecular level. However, there is no comparative data on the regulation of microRNAs (miRNAs) in visceral leishmaniasis (VL) and post-kala-azar dermal leishmaniasis (PKDL). In this current study, we compared the expression miRNA profile in host cells (G_THP_), with VL strain (G_VL_) and PKDL strain-infected host cell (G_PKDL_). Normalized read count comparison between different conditions revealed that the miRNAs are indeed differentially expressed. In G_PKDL_ with respect to G_VL_ and G_THP_, a total of 798 and 879 miRNAs were identified, out of which 349 and 518 are known miRNAs, respectively. Comparative analysis of changes in miRNA expression suggested that the involvement of differentially expressed miRNAs in various biological processes like PI3K pathway activation, cell cycle regulation, immunomodulation, apoptosis inhibition, different cytokine production, T-cell phenotypic transitions calcium regulation, and so on. A pathway enrichment study using *in silico* predicted gene targets of differentially expressed miRNAs showed evidence of potentially universal immune signaling pathway effects. Whereas cytokine–cytokine receptor interaction, phagocytosis, and transforming growth factor beta (TGF-β) signaling pathways were more highly enriched using targets of miRNAs upregulated in G_PKDL_. These findings could contribute to a better understanding of PKDL pathogenesis. Furthermore, the identified miRNAs could also be used as biomarkers in diagnosis, prognosis, and therapeutics of PKDL infection control.

## Introduction

Visceral leishmaniasis (VL) is a vector-borne disease caused by an intracellular parasite, *Leishmania donovani*. The highest burden of VL is reported from the states of Bihar, Jharkhand, West Bengal, and Uttar Pradesh of India. Among these, 90% of VL cases in India originate from Bihar (Singh et al., 2010). Post-kala-azar dermal leishmaniasis (PKDL), an enigmatic dermal sequel of VL, may appear after treatment of (VL, kala-azar) (Zijlstra et al., 2000). PKDL has several clinical polymorphic forms from a simple hypopigmented macular form of more developed lesions like papular, nodular, and mixed lesions. PKDL patients may play a role in the transmission of VL (Zijlstra et al., 2003; World Health Organization [WHO], 2010), which is a much-debated aspect. However, several cases have also been reported without a documented history of VL in 10–23% of patients (Zijlstra et al., 2017). Hence, it calls for better management of PKDL, especially susceptibility.

MicroRNAs (miRNAs) are small palindromic sequences, which are transcribed by RNA polymerase II or polymerase III to mature miRNA by Drosha and Dicer enzymes. The mature miRNA then binds to targeted RNA-induced silencing complex (RISC) and participates in the degradation of transcriptional silencing of messenger RNAs (Krol et al., 2010; Czech and Hannon, 2011). During the past years, an increasing amount of evidence indicates that miRNAs play an important role in many biological processes, like organ development, cell differentiation, proliferation, apoptosis, signal transduction, innate immunity, metabolism, disease pathogenesis, and tumorigenesis (Tiscornia and Belmonte, 2010; Huang et al., 2011; Zhang et al., 2014; Kumar and Lupold, 2016). Thus, an increased interest has developed for the subject.

“Next-generation sequencing” (NGS) is a high throughput technology allowing the generation and detection of thousands to millions of short sequencing reads in a single machine run, which can be rapidly introduced into clinical and public health laboratory practice (Besser et al., 2018). The profiling of miRNAs by NGS has progressed rapidly and is a promising field for applications in drug development (Liu et al., 2011). NGS is used to screen differential expression levels of microRNA between different disease conditions (Su et al., 2018).

In the field of *Leishmania*, Geraci et al. (2015) have reported that host cells after infection differentially expressed several miRNAs that may regulate different functions. Similarly, Tiwari et al. (2017) have identified 940 miRNAs in *L. donovani*-infected macrophages by *de novo* sequencing out of which levels of 85 miRNAs were found to be consistently modified by parasite infection. Singh et al. (2016) have demonstrated the novel regulatory role of host microRNA, MIR30A-3p in the modulation of host cell autophagy after infection with *L. donovani*. It was also reported that miRNAs can regulate immune signaling, cytokine production, and immune cell migration to control the VL infection in humans (Pandey et al., 2016).

Post-kala-azar dermal leishmaniasis, a cutaneous form of VL, commonly develops after apparent cure from VL. PKDL cases are treated according to national guidelines with miltefosine for 12 weeks; this regimen (50 mg twice per day) has a cure rate of 78%, but a higher dose showed a high cure rate but increased side effects (Mukhopadhyay et al., 2014). In this study, we are showing the differentially expressed miRNA in host cells infected with a PKDL strain (G_PKDL_) and that miRNAs may regulate different immunological pathways. The result of the current study may help in targeting the specific pathway, which may involve in PKDL disease progression. Targeting these pathways may help in future rational chemotherapeutic drug designing to counter drug resistance and the spread of PKDL.

## Materials and Methods

### *L. donovani* Promastigotes and Cell Line Maintenance Culture

*Leishmania donovani* isolates from a VL patient (MHOM/IN/83/AG83; repository number: RMRI/PB-0078) and from a PKDL patient (repository number: RMRI/PB-0088) were used in this study. Briefly, the collected splenic aspirates of VL were incubated in Roswell Park Memorial Institute (RPMI)-1640 medium (Gibco) (pH 7.4) supplemented with 10% fetal bovine serum (FBS) (Gibco) and 1% of penicillin (50 U/ml)–streptomycin (50 mg/ml) solution (Sigma) at 25°C. The amastigotes from splenic aspirates were transformed into promastigotes, and they were maintained further in RPMI-1640 medium supplemented with FBS. To obtain PKDL isolates, the nodular form of tissue was taken from the PKDL patients in RPMI-1640 media (Gibco, United States) with 20% FBS (Gibco, United States). Tissue cells containing amastigotes were converted into promastigotes form in RPMI-1640 media after 1–2 weeks. Promastigotes were subcultured in RPMI-1640 and maintained in tissue culture flask (Nunc) for infection study. Similarly, the human acute monocytic leukemia-derived THP-1 cell line procured from NCCS Pune was maintained in RPMI-1640 medium supplemented with 10% FBS at 37°C in a humidified mixture of 5% CO_2_ atmosphere for further studies.

### Infection of THP-1-Derived Macrophages

The VL and PKDL isolates were used for infection. Briefly, 10^4^ THP-1 cells/well were cultured in eight-well Lab-Tek chambers (Nunc, Roskilde, Denmark). To differentiate monocyte into a macrophage, cells were treated with phorbol myristate acetate (PMA, Sigma-Aldrich) at 40 ng/ml for 24 h (37°C, 5% CO_2_). After 24 h, cells were gently washed with warm medium, replenished with fresh medium, and incubated for an additional 24 h. Parasites [at a multiplicity of infection (MOI), ∼10:1] of both VL and PKDL isolates of *L. donovani* were added into the THP-1-derived macrophage cultures and maintained at 37°C in 5% CO_2_ overnight. After 12 h, non-internalized promastigotes were eliminated. Infected cells were collected by washing with phosphate-buffered saline (PBS; pH 7.2).

### RNA Isolation

RNA isolation from samples THP-1 (G_THP_), THP-1 infected with VL parasites (G_VL_), and THP-1 infected with PKDL parasites (G_PKDL_) was performed using Ambion mirVANA miRNA isolation kit (Cat-AM1560, as per manufacturer’s instructions). Briefly, cells were lysed with 300 μl of lysis/binding buffer. Homogenate additive was added to the lysate at 1/10th of the lysate volume followed by the addition of acid phenol–chloroform. After brief vortex and centrifugation, the upper aqueous phase was aspirated into a new vial. This aqueous phase was mixed with 1.25 volumes of 100% ethanol and loaded onto a filter cartridge. The remaining steps of the purification were followed as per the manufacturer’s guidelines, including on-column DNase treatment Qiagen (Cat-79254). RNA was eluted in 25 μl of nuclease-free water (Ambion, Cat-AM9938). Eluted RNA was stored at −80°C until further use. The concentration and purity of the RNA extracted were evaluated using the Nanodrop spectrophotometer (Thermo Scientific; 2000). The integrity of the extracted RNA was analyzed with the 2100 expert Bioanalyzer (Agilent). We considered RNA to be of good quality based on the 260/280 values (Nanodrop) and optimal RNA integrity profile in the Bioanalyzer.

### Library Preparation and Sequencing

Small RNA sequencing libraries were prepared using NEXTFLEX Small RNA-Seq Kit v3 (Bio Scientific Corp., TX, United States) using the manufacturer’s protocol. Briefly, 500 ng of total RNA was used as starting material. 3′ adapters were ligated to the specific 3′OH group of microRNAs followed by ligation of 5′ adapter. Adapter-ligated fragments were reverse transcribed with Moloney murine leukemia virus (M-MuLV) reverse transcriptase by priming with reverse transcription primers. Complementary DNA (cDNA) thus formed was enriched and indexed by PCR (15 cycles). Libraries were size selected using the NEXTflex magnetic bead purification system and finally reconstituted in nuclease-free water. The sequencing libraries were quantified by the Qubit fluorometer (Thermo Fisher Scientific, United States), and its fragment size distribution was analyzed on Tape Station using Agilent D1000 Screen Tapes (Agilent Technologies, United States). The libraries were sequenced on Illumina NextSeq 500 sequencer (Illumina, Inc., United States) for 75-bp single read chemistry following manufacture’s guidelines.

### Raw Data Generation and Filtration

In this study, two infected human cell lines (G_VL_ and G_PKDL_) and one uninfected cell line (G_THP_) were sequenced in NGS single-end read chemistry and carried out analysis against *Homo sapiens* GRCh38 genome data. The raw data of 75 bp length was generated on the Illumina platform and received in FASTQ format. srna-workbenchV3.0_ALPHA was used to trim the adapter and performed length filtering (minimum length of 16 bp and maximum of 40 bp). The low-quality and contaminated reads were removed on the following criteria to obtain final clean reads: (i) elimination of low quality reads (<q30), (ii) elimination of reads without 3′ adapters, (iii) elimination of reads with 5′ adapters, (iv) elimination of reads without insert, (v) elimination of reads <16 and >40 bp, (vi) elimination of reads not matching to reference genome, (vii) elimination of reads matching to other non-coding RNAs (ncRNAs) [ribosomal (rRNA), transfer (tRNA), small nuclear (snRNA), PIWI-interacting (piRNA), and small nucleolar RNAs (snoRNAs)]. Sequences ≥16 and ≤40 bp length were considered for further analysis (Supplementary Figure S1).

### Identification of Known (Conserved) miRNA

miRNAs were identified by a homology approach against matured human miRNAs. The chromosomal location of previously known miRNAs can be obtained from the miRbase^1^. All the sequences were aligned to *H. sapiens* GRCh38 build genome using bowtie-1.1.12. Aligned reads were extracted and checked for other ncRNA (rRNA, tRNA, snRNA, snoRNA, and piRNA) contamination. The unaligned reads to ncRNAs were used for known miRNA prediction. Reads were made unique, and hence, read count profile was generated. Furthermore, a homology search of these miRNAs was done against human mature miRNA sequences retrieved from miRbase-21 (Kozomara and Griffiths-Jones, 2014) using NCBI-BLAST 2.2.30 (Altschul et al., 1990) at e−4 and non-gapped alignment (Supplementary Figure S1).

### Identification of Novel miRNA

Sequences not showing hits with known miRNAs were extracted and were considered for novel miRNA prediction. These reads were aligned to the reference genome using bowtie. Novel miRNAs were predicted from the aligned data using Mireap_0.22b (https://github.com/liqb/mireap). To identify true novel miRNAs, predicted candidate miRNAs were matched against human miRNAs predicted in the genomes but not present in miRBase. Unaligned predicted miRNAs were considered as potential novel miRNAs if the predicted secondary structure is a proper stem-loop structure defined for an miRNA (Supplementary Figure S1).

### Differential Expression Study

Read count table for all the samples was generated. Differential gene expression (DGE) analysis was carried out using the DESeq (Anders and Huber, 2010) tool. Variations in the reads are normalized by the library normalization method opted from the DESeq library. DESeq calculates the size factor; each read count is normalized by dividing with size factor. Mean normalized read counts of the samples in a given condition are used for DGE calculation and heatmap. For regulation calculation between the comparisons, log2fold of 1 was used as a cutoff. Expression values of miRNAs are DESEq normalized values between samples 1 and 2. Normalized expression of a given miRNA may change with respect to the comparison sample and their library size. Fold changes are calculated based on “expression of treated sample/expression of control sample.” miRNAs >1 were considered as “UP” regulated, miRNAs <−1 were considered as “DOWN,” and those between 1 and −1 were flagged as “NEUTRAL” (Supplementary Figure S1 and Supplementary File S1).

### Pathway Overrepresentation Analysis of miRNA Targets

To analyze the overrepresentation of miRNA–gene association in selected immunological pathways, miRNA Enrichment Analysis and Annotation tool (miEAA) (Backes et al., 2016) was used. For each analysis, corresponding miRNAs (upregulated, downregulated, and only expressed in each group) as miRBase IDs (Kozomara et al., 2019) were given as test set (G_PKDL_), and all the miRNAs expressed in the corresponding group were used as reference set (G_THP_ and G_VL_). A significance level of 0.05 [false discovery rate (FDR) corrected *P*-value] and a threshold of 2 (observed miRNA) was chosen for all the analysis. From the obtained results, the ratio between the observed and expected number of miRNA (for each chosen pathway) was computed for selected pathways using in-house Perl script. All the graphs based on sequencing data were generated using R programming environment.

### miRNA–Target Network Construction

miRNet^2^ (Fan and Xia, 2018) was used for constructing miRNA–gene target network. Only the upregulated miRNAs of G_PKDL_ were queried. Interactions reported by crosslinking immunoprecipitation (CLIP) were alone considered for the network generation; all other interactions were removed. The targets in the network were chosen using Reactome knowledgebase (Fabregat et al., 2018) with an algorithm set as a hypergeometric test and with a statistical threshold of *P* < 0.001. In PKDL, the Th_2_ response shows the presence and persistence in the skin. Therefore, we are trying to find the networking molecule regulated by miRNAs, which are responsible for modulating the Th_2_-responsive genes.

### IL-10 ELISA

THP cells (1 × 10^6^) were uninfected and infected with 1 × 10^7^ late log phase *L. donovani* promastigotes (1:10) isolates from VL patients for 12 h. In another set, THP cells were infected with 1 × 10^7^ late log phase *L. donovani* promastigotes (1:10) isolates from PKDL patients for 12 h. The culture supernatants after incubation were collected and tested for cytokines interleukin (IL)-10 using sandwich ELISA kit (R&D system, United States) according to the manufacturer’s protocol. The detection limit of these assays was <5.3 pg/ml for IL-10, respectively. Results are represented as mean ± SD for three independent experiments.

### Luciferase Reporter Assay

We predicted the target of miRNA-93-5p by online software Target Scan based on target rank and score. The miRNA-93-5p targets TGFBR2 gene, which has a crucial role in immune responses during visceral leishmaniasis. Binding site of miRNA-93-5p and TGFBR2 gene was detected by Target Scan 7.2. The 3′-untranslated region (UTR) of genes of interest containing the putative miRNA target site(s) WT-TGFBR2 gene (wild-type) and Mut-TGFBR2 gene (mutant) was cloned into the *Xba*I site of the pGL3 control vector containing firefly luciferase gene linked to the 3′-UTR of the gene (Promega, United States). Macrophages were transfected by constructed pGL3 vector with either controls or mimics of miRNA-93-5p Lipofectamine 2000 (Invitrogen, United States). After 24 h, cells were lyzed, and luciferase activity was measured by the dual-gloluciferase assay system (Promega, United States) according to manufacturer’s instruction on luminometer. Firefly luciferase reading was normalized by the renilla luciferase reporter.

### Evaluation of the Role of miRNA-93-5p in TGFBR2 Mediated Immune Responses

The role of miRNA-93-5p in TGFBR2-mediated proinflammatory immune responses was evaluated by silencing with antagomir. The miR-93-5p antagomir was designed in our laboratory and was synthesized by Integrated DNA Technologies, New Delhi, using the sequence, CUACCUGCACGAACAGCACUUUG, with the following modifications: the 2′-OMe-modified bases (2′-hydroxyl of the ribose was replaced with a methoxy group), phosphorothioate (phosphodiester linkages were changed to phosphorothioate) on the first two and last four bases, and an addition of cholesterol motif at 3′ end through a hydroxyproline-modified linkage. Antagomir scramble (mismatched miR-93-5p) was also obtained from the same company and used as negative control. The expression of miR-93-5p was silenced by antagomir according to the standard protocol described previously with required modification. Briefly, macrophages (10^6^ cells/well) were suspended in 500 μl of serum-free media for 2 h at 37°C and 5% CO_2_ supplemented with 1 mM of antagomir-93-5p or antagomir scramble. After incubation, 150 μl of media containing serum and antibiotics was added and cultured for 24 h in a CO_2_ incubator. For evaluating the functional role of miR-93-5p, four experimental groups were plotted as follows: (1) uninfected Mφ, (2) Mφ infected with *L. donovani*, (3) miR-93-5p silenced Mφ infected with *L. donovani*, and (4) antagomir scramble-treated Mφ infected with *L. donovani*. Macrophages were incubated in the CO_2_ incubator with 5% CO_2_ for 24 h. After 24 h of incubation, supernatants were collected for the measurement of cytokines.

### Statistical Analysis

Statistical analysis of the differences between means of groups was performed with two-tailed Student’s *t*-test and ANOVA on GraphPad Prism 5.0 software. A *P* < 0.05 was considered significant, and a *P* < 0.01 was considered highly significant.

## Results

### Confirmation of Infection Study

**FIGURE 1:**
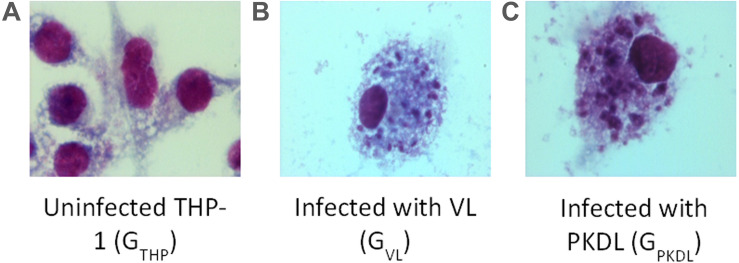
Intracellular *Leishmania donovani* amastigotes visualized by stain. **(A)** Uninfected control THP cell. **(B)** THP cell infected with visceral leishmaniasis (VL) strain (G_VL_). **(C)** THP cell infected with post-kala-azar dermal leishmaniasis (PKDL) strain (G_PKDL_). G_VL_ and G_PKDL_ showed 60–70% infection rate. Optical microscopy at 100× oil immersion.

Promastigotes culture of VL and PKDL strains were used for the infection of the human THP-1 cell line. Infection was confirmed by Giemsa staining (Merck) in G_VL_ and G_PKDL_ infection compared to the G_THP_ groups. At least 100 cells were counted manually for each condition to determine the average number of parasites per macrophage. There were 62 and 64% infected G_VL_ and G_PKDL_, respectively. Examples of infected cells are shown in Figure 1.

### Quality Control of RNA Preparation

**FIGURE 2:**
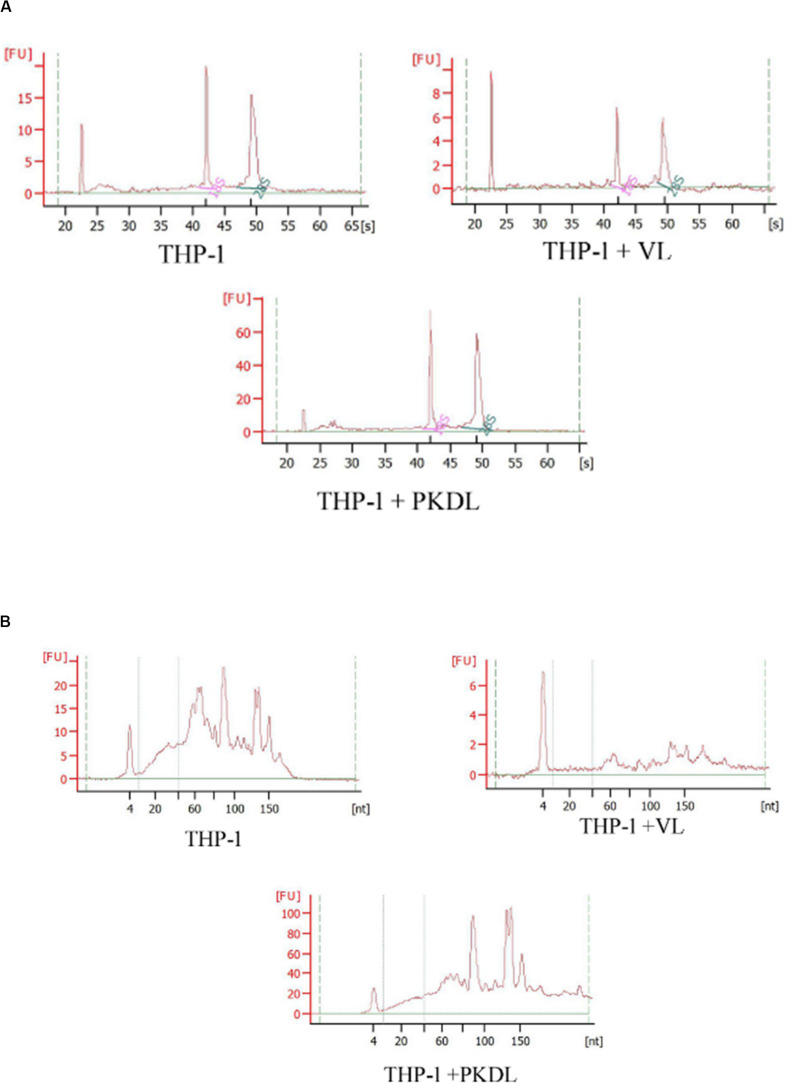
RNA integrity assessment based on the ratio of 28S:18S rRNA, estimated from the **(A)** band integrity or **(B)** densitometry plot.

As the quality and integrity of RNA was very crucial for this study, we first evaluated these qualities of the RNA yielded in all experimental infection. Intact eukaryotic total RNA should yield clear 28S and 18S rRNA bands. The 28S rRNA band is approximately twice as intense as the 18S rRNA band (2:1 ratio). Since the human interpretation of gel images is subjective and is inconsistent, we used RNA integrity number (RIN) analysis. Agilent has developed a software algorithm that allows for the calculation of an RIN from a digital representation of the size distribution of RNA molecules (which can be obtained from an Agilent Bioanalyzer). The RIN is based on a numbering system from 1 to 10, with 1 being the most degraded and 10 being the most intact. This approach facilitates the interpretation and reproducibility of RNA quality assessments and provides a means by which samples can be compared in a standardized manner (Figures 2A,B).

### miRNA Differential Expression

**FIGURE 3:**
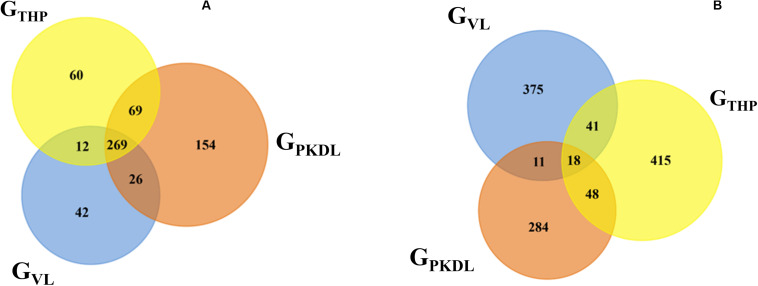
Venn diagram showing the differentially expressed overlaps of microRNA (miRNA). **(A)** Overlaps of known miRNAs between G_THP_ (THP-1), G_VL_ [visceral leishmaniasis (VL)], and G_PKDL_ [post-kala-azar dermal leishmaniasis (PKDL)] groups. **(B)** Overlaps of novel miRNAs between G_THP_, G_VL_, and with G_PKDL_ groups.

**FIGURE 4:**
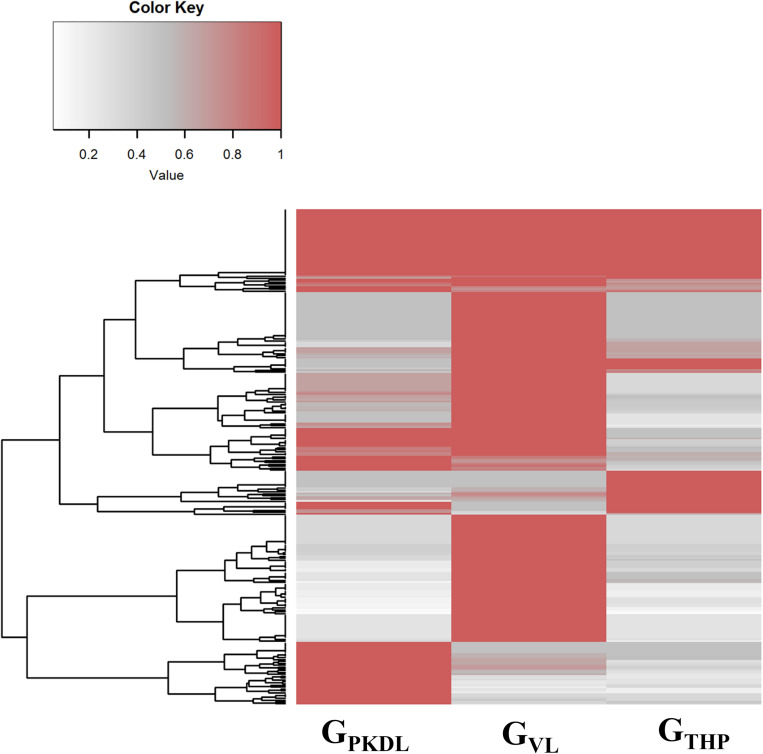
Commonly expressed microRNAs (miRNAs) of G_THP_ (THP-1), G_VL_ [visceral leishmaniasis (VL)], and G_PKDL_ [post-kala-azar dermal leishmaniasis (PKDL)] and their corresponding normalized read counts.

Here, we have identified the differentially expressed known and novel miRNA of G_THP_, G_VL_, and G_PKDL_ groups. NGS analysis confirmed that a total of 935 miRNAs were identified in G_THP_, out of which 410 are known miRNAs and 525 are novel miRNAs. With respect to G_VL_ and G_PKDL_, a total of 798 and 879 miRNAs were identified, out of which 349 and 518 are known miRNAs, respectively. The overlaps of known and novel miRNAs between each group were indicated as the Venn diagram (Figure 3A). It could be observed that a total of 269 known miRNAs was expressed in all three conditions. To infer the difference in miRNA read count between the common identified 269 miRNAs, the read counts of each miRNA were normalized and plotted (Figure 4). The heat map indicates that the read count profile of each group is different. When novel miRNAs were discussed, we identified the 415 novel miRNAs for G_THP_, 375 novel miRNAs exclusively for G_VL_, and 284 novel miRNAs exclusively for G_PKDL_ groups. After the Venn diagram analysis, we observed that 18 novel miRNAs are commonly found in all three conditions (Figure 3B).

### PKDL Responsive Upregulated miRNAs

**FIGURE 5:**
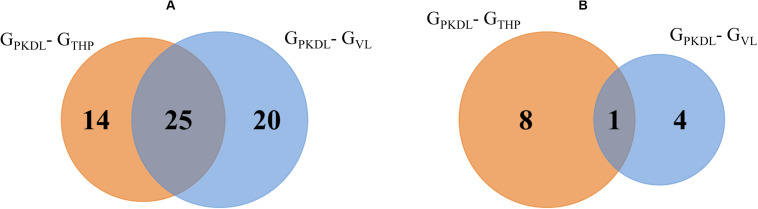
Venn diagram shows the G_PKDL_ upregulated microRNAs (miRNAs). **(A)** Overlaps of known upregulated miRNAs of G_PKDL_ with respect to G_THP_ and to G_VL_. **(B)** Overlaps of novel upregulated miRNAs of G_PKDL_ with respect to G_THP_ and G_VL_.

To identify the differentially overexpressed miRNAs in G_PKDL_ with respect to G_THP_ and G_VL_, we subjected our results to DESeq2 (DGE) analysis:

(A) To get separate information about known miRNAs, a total of 39 known miRNAs in G_PKDL_ with respect to G_THP_ and a total of 45 miRNAs (with respect to G_VL_) were significantly upregulated in G_PKDL_ (Figure 5A). Moreover, a total of 25 miRNAs are commonly upregulated in both G_THP_ and G_VL_ groups, while a total of 14 and 20 miRNAs are upregulated only in G_THP_ and G_VL_, respectively. The families of miRNA groups are represented in Table 1C and Supplementary Figure S2.

**TABLE 1:** List of up- and downregulated novel and known miRNAs.

G_THP_ only	G_VL_ only	Common
**(A) Downregulated novel miRNAs sequence**		
CCGGCCGGCCGGGCGCCGAG	CACCCGGCGGGGCGGGGCGGGG	CCGCCCCCCGGCGGCGGCGCC
CGCTCGTGGCGGTCCTGCAGCG	TCGCCAGGCCCGGCGGGGCCCC	CGCCCGGCGGCGGCGGCGGCG
GACGCCGGCGGCGGCGGGCTCC		GCCCGGCTGGGCGCGGCGCT
GAGCCGGCGGCGGCGGGCAGCG		GCGGGGGTTCCTCCCTGGGGCAC
GCCACGGCGGCGGCGCCTCC		
GCGGCCGCGCCGGCCGGGGCGC		
GGCACAGGCGCGGGTATCTCA		
TCGGGGTTCCTGCCGGGAGGC		
**(B) Upregulated novel miRNAs sequence**		
AACCCGCGACCTCAGATCCCCAG	CACCCAGGAGTGGAGGTTGC	CTCCCATTAGAGGGCACTCCC
CACGTGTGCGGAGTGCCCCGC	CCAGCCTCCGGCGGGTGTTCC	
CCTCAGCTCAGGGAGGGCAGT	CCTGAGCTCAGGGAGCTCAC	
CGGACAGCTTCCGGGATGGGA	GATCCCTCGTGGGGGGATGG	
CTTCACGGAAGGGCAGGCTT		
GCGCGTGAAGGGCGGGTAGTTG		
TGCTATGCGTGGGAGGGGCTC		
TTACAGTTTGACTGGGGCAGTGT		
**(C) Upregulated known miRNAs**		
hsa-miR-10400-5p; hsa-miR-143-3p; hsa-miR-15a-5p; hsa-miR-17-5p; hsa-miR-181a-5p; hsa-miR-186-5p; hsa-miR-18a-5p; hsa-miR-195-5p; hsa-miR-19a-3p; hsa-miR-19b-3p; hsa-miR-26b-5p; hsa-miR-29a-3p; hsa-miR-335-5p; hsa-miR-454-3p	hsa-let-7b-5p; hsa-let-7f-2-3p; hsa-let-7g-5p; hsa-miR-12136; hsa-miR-15b-3p; hsa-miR-191-5p; hsa-miR-221-5p; hsa-miR-27b-3p; hsa-miR-301a-3p; hsa-miR-30c-5p; hsa-miR-30e-3p; hsa-miR-324-5p; hsa-miR-326; hsa-miR-339-5p; hsa-miR-450b-5p; hsa-miR-576-3p; hsa-miR-663b; hsa-miR-769-5p; hsa-miR-96-5p; hsa-miR-98-5p	hsa-let-7c-5p; hsa-let-7f-5p; hsa-miR-101-3p; hsa-miR-103b; hsa-miR-106b-5p; hsa-miR-146a-5p; hsa-miR-155-5p; hsa-miR-16-5p; hsa-miR-21-5p; hsa-miR-221-3p; hsa-miR-26a-5p; hsa-miR-27a-3p; hsa-miR-30a-5p; hsa-miR-30e-5p; hsa-miR-3141; hsa-miR-3184-5p; hsa-miR-374a-5p; hsa-miR-374c-3p; hsa-miR-425-5p; hsa-miR-542-3p; hsa-miR-619-5p; hsa-miR-7-5p; hsa-miR-9-3p; hsa-miR-9-5p; hsa-miR-93-5p
**(D) Downregulated known miRNAs**		
hsa-miR-132-3p; hsa-miR-24-2-5p; hsa-miR-503-5p; hsa-miR-99b-5p	hsa-let-7b-3p; hsa-let-7d-3p; hsa-let-7i-3p; hsa-miR-10396a-3p; hsa-miR-1246; hsa-miR-1249-3p; hsa-miR-1260b; hsa-miR-1269b; hsa-miR-1301-3p; hsa-miR-1303; hsa-miR-145-5p; hsa-miR-146b-3p; hsa-miR-148b-5p; hsa-miR-16-2-3p; hsa-miR-181a-2-3p; hsa-miR-181d-5p; hsa-miR-193a-5p; hsa-miR-212-3p; hsa-miR-218-5p; hsa-miR-2355-3p; hsa-miR-25-5p; hsa-miR-30c-1-3p; hsa-miR-3128; hsa-miR-3177-3p; hsa-miR-3196; hsa-miR-330-5p; hsa-miR-342-5p; hsa-miR-3609; hsa-miR-361-3p; hsa-miR-365b-3p; hsa-miR-374a-3p; hsa-miR-4454; hsa-miR-4466; hsa-miR-455-5p; hsa-miR-4742-5p; hsa-miR-484; hsa-miR-501-3p; hsa-miR-548at-5p; hsa-miR-548t-3p; hsa-miR-574-3p; hsa-miR-625-3p; hsa-miR-6501-3p; hsa-miR-92a-1-5p; hsa-miR-941; hsa-miR-9901	hsa-miR-125a-5p; hsa-miR-7704

(B) Similarly, when we focus on novel miRNAs, then we observed eight and four novel miRNAs that are exclusively found in G_THP_ and G_VL_, respectively, and one miRNA common among these two conditions (Figure 5B). The sequences of novel miRNAs are represented in Table 1B.

### PKDL-Responsive Downregulated miRNA

**FIGURE 6:**
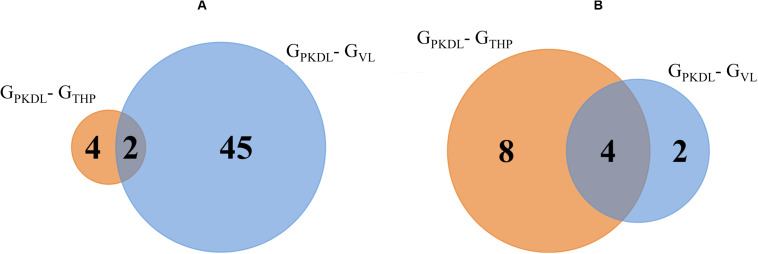
Venn diagram shows the post-kala-azar dermal leishmaniasis (PKDL) responsive downregulated microRNA (miRNA). **(A)** Overlaps of known downregulated miRNAs of G_PKDL_ with respect to G_THP_ (PKDL–THP-1) and G_VL_ (PKDL–visceral leishmaniasis (VL)]. **(B)** Overlaps of the novel downregulated miRNAs of G_PKDL_ with respect to G_THP_ (PKDL–THP-1) and G_VL_ (PKDL–VL).

Analysis of known miRNAs that are downregulated in G_PKDL_ condition with respect to G_THP_ and G_VL_ conditions were performed. We observed that two downregulated miRNAs are common to G_THP_ and G_VL_; four miRNAs exclusively downregulated in G_THP_ and 45 miRNAs exclusively downregulated in G_VL_ (Figure 6A and Table 1D). For novel miRNAs, four miRNAs were commonly observed with G_THP_ and G_VL_; eight miRNAs exclusively downregulated in G_THP_ and two miRNAs exclusively downregulated in G_VL_ (Figure 6B and Table 1A).

### Target Pathway Prediction for Differentially Expressed miRNAs

**FIGURE 7:**
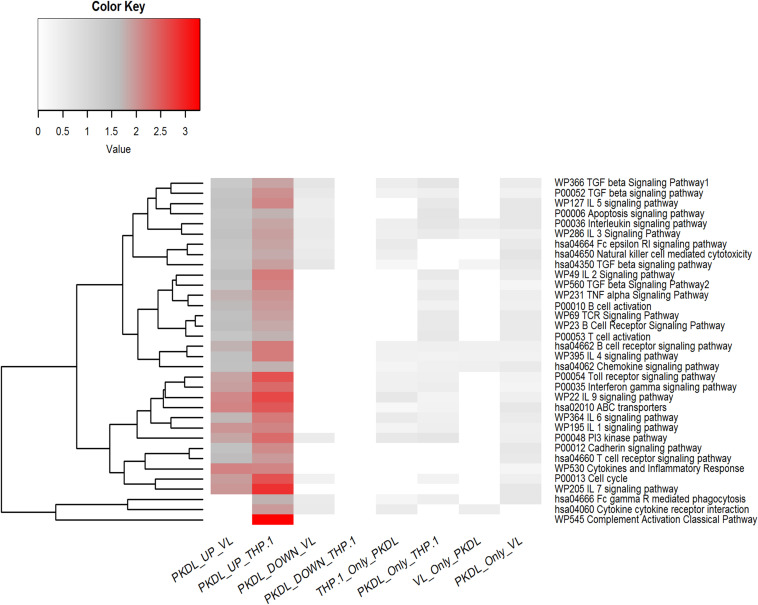
Pathway enrichment test for differentially expressed microRNAs (miRNAs) (up- and downregulated in G_PKDL_ with respect to G_THP_ and G_VL_) and unique miRNAs of G_THP_, G_VL_, and G_PKDL_. Horizontal lanes are marked with selected immunological pathways, and vertical lanes are marked with comparison groups. The enrichment analysis [false discovery rate (FDR) corrected *P* < 0.05] for each comparison groups are performed separately with reference set being all expressed miRNAs of the corresponding comparison groups.

All the differentially expressed miRNAs (up- and downregulated miRNA in G_PKDL_ with respect to G_THP_ and G_VL_) were subjected to pathway overrepresentation analysis using the miEAA tool. A total of 35 pathways related to immunological response were selected for the analysis. The expected number of miRNAs and observed number of miRNAs having an miRNA–gene association in selected immunological pathways for each group were analyzed, and the ratio between them was plotted (Figure 7). The result indicates that the miRNAs upregulated in G_PKDL_ (with respect to both G_THP_ and G_VL_group) are significantly overrepresented in the selected immunological pathways. Interestingly, the downregulated miRNAs and other miRNAs present only in the corresponding groups are underrepresented in the selected immunological pathways. In addition, a marked difference of overrepresentation was observed between G_PKDL_ upregulated miRNAs of G_THP_ and G_VL_ for three pathways, viz., “hsa04666 Fc gamma R mediated phagocytosis,” “hsa04060 cytokine receptor interaction,” and “P0052 TGF-β signaling pathway.” The miRNA–gene associations of those pathways are overrepresented in G_THP_, while in G_VL_, there was no single miRNA associated with genes of the corresponding three pathways. Studies on the involvement of certain immunological factors such as some cytokines [IL-10, transforming growth factor beta (TGF-β), interferon-gamma (IFN-γ), etc.] and chemokines [macrophage inflammatory protein (MIP-1), regulated upon activation, normal T cell expressed, and secreted (RANTES), etc.] in the dermal lesions of PKDL cases (before and after chemotherapy) may provide insight for any role in the disappearance of lesion and treatment response. The values in the heat map indicate the number of miRNAs having putative gene targets involved in the corresponding pathway.

### miRNAs Regulating TGF-Beta Mediated Signaling Pathway in PKDL

**FIGURE 8:**
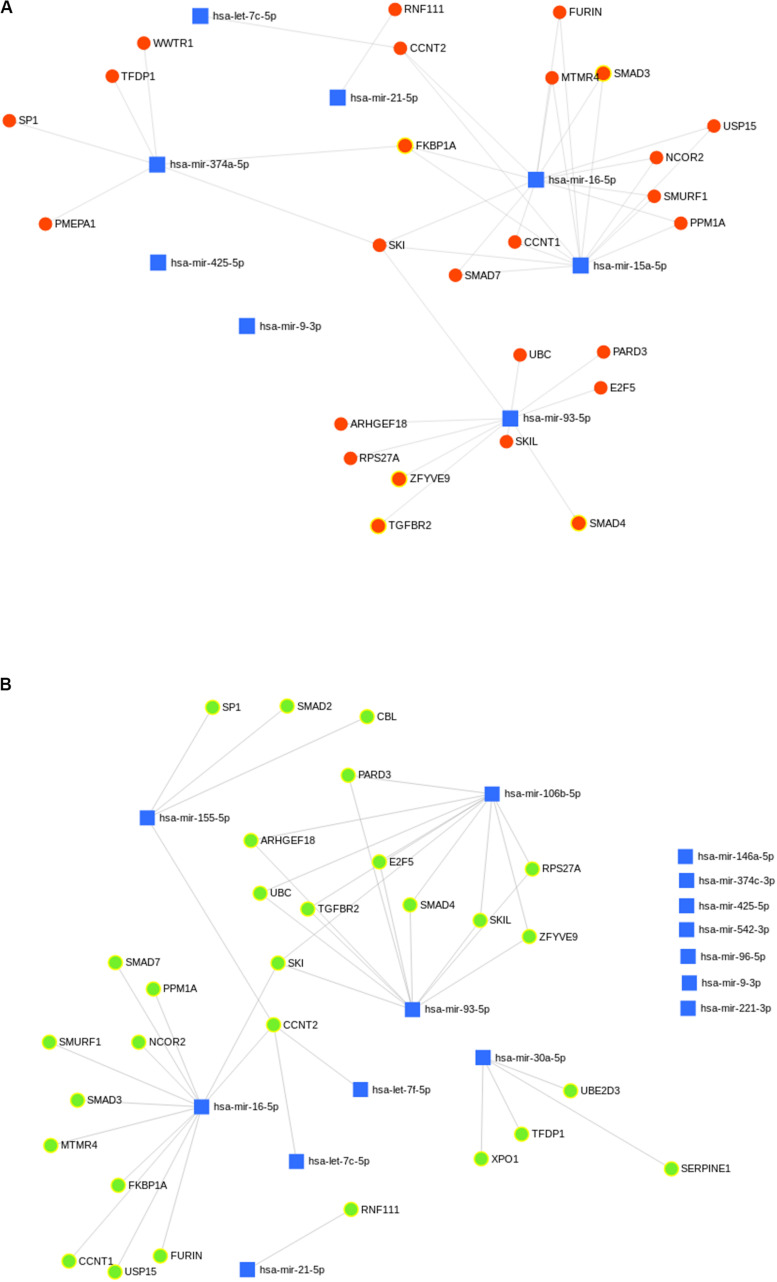
**(A,B)** Predicted biological processes of upregulated microRNA (miRNA) in G_PKDL_ with respect to **(A)** G_THP_ and **(B)** G_VL_. The interlinked miRNAs and important pathways linked to macrophage effector functions. The interaction of upregulated miRNAs and their targeted biological processes. The highlighted red and green colors denote interlinked miRNAs with key biological processes relevant to macrophage dysfunction such as cytokine response, immune response, etc.

A total of 29 and 26 gene targets belonging to the TGF-beta signaling pathway were mapped to eight and six miRNAs of G_THP_ and G_VL_, respectively (Figures 8A,B). Eight and six miRNAs upregulated in G_PKDL_ compared to G_THP_ and G_VL_, respectively. Whereas in G_VL_, out of the 15 upregulated miRNAs, only 8 had one or more gene targets belonging to the TGF-beta signaling pathway. A total of 24 gene targets were commonly observed among the upregulated miRNAs of G_THP_ and G_VL_. Gene targets CBL, SERPINE1, SMAD2, UBE2D3, and XPO1 were only observed in the G_VL_ pool, and gene targets PMEPA1 and WWTR1 were only observed in G_THP_.

### IL-10 ELISA

**FIGURE 9:**
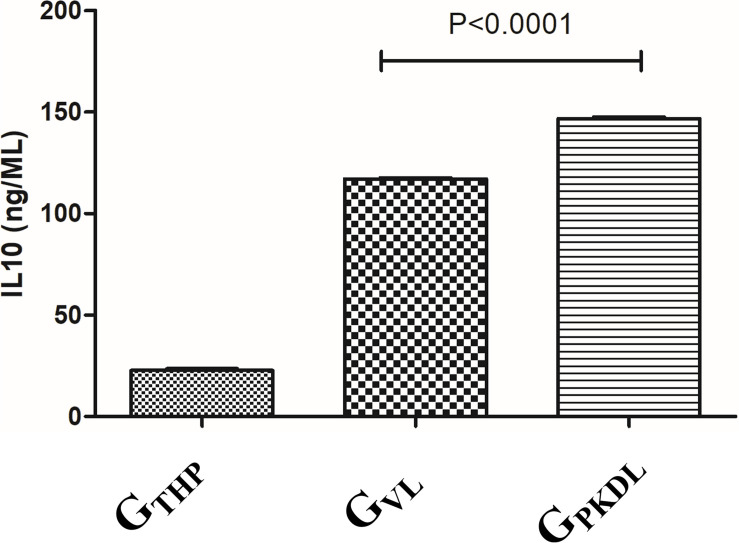
THP cells (1 × 10^6^) were uninfected and infected with 1 × 10^7^ late log phase *L. donovani* promastigotes (1:10) isolates from visceral leishmaniasis (VL) patients for 12 h. In another set, THP cells were infected with 1 × 10^7^ late log phase *L. donovani* promastigotes (1:10) isolates from post-kala-azar dermal leishmaniasis (PKDL) patients for 12 h. Results are represented as mean ± SD for three independent experiments.

The immune profile in PKDL cases as well as VL cases was remarkably dissociated mostly; however, due to some differences in immune paradigm, skin rash prevails without systemic illness (Zijlstra et al., 2003). In contrast to the above facts, we have observed ∼1.2-fold production of IL-10 in G_PKDL_ when compared to G_VL_ in the culture supernatant (Figure 9).

### Luciferase Reporter Assay

**FIGURE 10:**
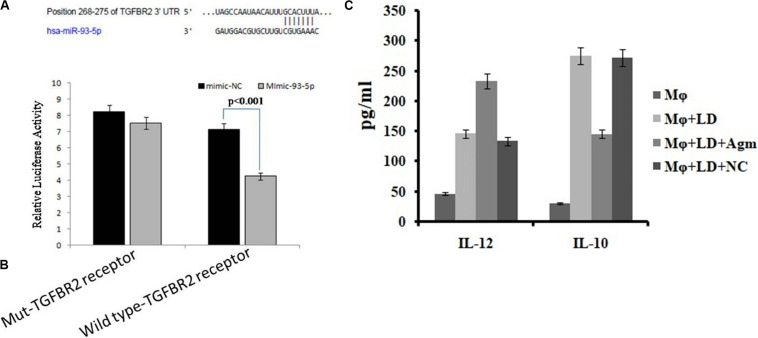
Prediction and validation of TGFBR as target gene of miRNA-93-5p. **(A)** Schematic representation of miRNA-93-5p sequence bound to TGFBR receptor. The luciferase constructs fused to 30 untranslated regions (UTRs) (or Mut) of TGFB receptor were cotransfected in macrophages cells with mimic miRNA-93 or mimic-NC. The luciferase activities were expressed as the ratio of firefly luciferase over renilla luciferase. **(B)** The macrophages transfected with mimic of miRNA-93 significantly reduced the luciferase activities (*P* < 0.001) of genes fused to the TGFB receptor family 30 UTR. **(C)** Effect of antagomir miRNA-93-5p in the activation of interleukin (IL)-10/IL-12 secretion from macrophages after infection.

We found that miR-93-5p targets TGFR2 gene, which plays an important role in immune responses during visceral leishmaniasis. Furthermore, for validation of miR-93-5p target genes, we performed the luciferase reporter assay. The transfection with mimic of miR-93-5p significantly (*P* < 0.001) reduced the luciferase activities of genes fused to TGFBR2 3′-UTR. Mutated 3′-UTR seed sequences of TGFBR2 did not show any significant luciferase activities treated with mimic of miR-93-5p (Figures 10A,B).

### Estimation of Cytokines

The release of cytokines in cultured supernatant was measured by cytokine ELISA and is depicted in Figure 10C. The level of proinflammatory cytokines, i.e., IL-12, was significantly higher (*P* < 0.05) in antagomir-treated macrophages compared to untreated macrophages. However, the level of anti-inflammatory cytokines, i.e., IL-10, in antagomir-treated macrophages was found to be lower (*P* < 0.001) compared to that in untreated macrophages. The macrophages treated with antagomir scramble did not show significant change in pro- or anti-inflammatory cytokine level compared to untreated macrophages. These results indicated that miR-93-5p has an important role in IL-10 cytokine production during *Leishmania* infection, and its control can upregulate the production of inflammatory cytokines.

## Discussion

Change in the dynamic cellular environment after infection of any organism depends on the various factors of the cells, and one of the important factors is microRNA. MicroRNA profiling of human macrophage THP-1 cell line infected with *L. donovani* has revealed enormous information leading to the identification of several key miRNAs that target several immunological signaling cascades (Tiwari et al., 2017). In this study, we have, for the first time, shown the unique microRNA that is exclusively differentially expressed in human macrophage THP-1 cell line after infection with *L. donovani* isolated from PKDL patients (G_PKDL_) compared with uninfected THP-1 (G_THP_) and THP-1 infected with *L. donovani* isolated from visceral leishmaniasis patients (G_VL_) by NGS and their role in immunological pathway regulation.

Next-generation sequencing has become a popular means for miRNA profiling and has led to the identification of 150 known miRNAs and 415 novel miRNAs using the *de novo* sequencing approach, in which expression levels were largely altered in G_PKDL_. miRNAs are known to regulate the expression of target genes both at the levels of messenger RNA (mRNA) translation and mRNA stability, leading to a negative correlation between expression levels of these master regulators and their target mRNAs. Furthermore, the *in silico* analysis acknowledged the role of the immunological pathway that possibly facilitates parasite survival through differential regulation of macrophage effector genes.

As we know, PKDL is a dermal condition that, in Asia, occurs in patients after VL treatment and without any prior infection of VL (Mukhopadhyay et al., 2014). Immune responses in leishmaniasis are often described in terms of a (T helper) Th_1_ and Th_2_ response. Elevation of IL-10 and TGF-β probably reflects the role of these cytokines in reactivation of the disease in the form of PKDL (Ismail et al., 1999). TGF-β, TNF-α, IL-10, and IL-12 produced by keratinocyte plays a major role in disease progression while the expression of IFN-γR and TNFR1 was lower in patients with PKDL in India and increased after treatment (Ansari et al., 2006, 2008). It was also reported that, in the PKDL lesion, the upregulation of IL-17 induces TNF-α and NO level (Mukhopadhyay et al., 2014). Biological pathway enrichments were performed using all potential *in silico*-predicted targets of up- or downregulated miRNAs, regulating the immune responses either by different cytokine regulation, T-cell regulation, TGF-β signaling pathway, Toll-like receptor (TLR)-mediated regulation, ABC transporter regulation, cell cycle analysis, PI3K pathway, etc. in G_PKDL_ with respect to G_THP_ and G_VL_. Our analysis showed that miRNAs may reciprocally regulate these pathways and help the parasites in pathogenesis.

The results of the present study demonstrate the regulation of IL-10 as well as of TGF-β in PKDL by different miRNAs, which adds vital information on the mechanism of disease pathogenesis, implicating a role for these cytokines in derailing Th_1_ responses. Interleukin-10 has a major role in the pathogenesis of VL and PKDL (Ismail et al., 1999). Differences in serum IL-10 levels in VL and PKDL cases provide clue for disease profile and parasite behavior. In a previous study, increased IL-10 is regarded as hallmark of Indian PKDL, as they found IL-10 production enhanced as in PKDL cases as compared to VL cases (Ganguly et al., 2008). Accordingly our results also showed high level of IL-10 and highlighting the importance of the dynamics of the cytokine profiles between VL and PKDL. miRNA levels play an important role in regulating macrophage functions during infection (Lemaire et al., 2013). Several studies have shown the importance of IL-10 and TGF-β in different types of leishmaniasis. In mouse and human studies, both IL-10 and TGF-β have been associated with activation/differentiation of Treg cells (Maloy and Powrie, 2001), which regulate Th_1_ and Th_2_ responses. Moreover, Tregs can suppress the activation/maturation, expansion, and differentiation of a variety of immune cell types via several mechanisms, including secretion of anti-inflammatory cytokines like IL-10 and TGF-β, and sequestering IL-2 (Levings et al., 2006). T regulatory cells (Tregs) are a proportion of T cells usually identified by the expression of the transcription factor FoxP3 and a high level of expression of CD25 and in Indian PKDL, an increased presence of forkhead box P3 (FoxP3), a key molecular marker of Tregs (Ganguly et al., 2010). In PKDL, enhanced secretion of IL-10 and TGF-β by antigen-stimulated peripheral blood mononuclear cells (PBMCs) has been associated with disease severity (Saha et al., 2007). However, TGF-β signaling pathways were among the top 10 most significantly enriched Kyoto Encyclopedia of Genes and Genomes (KEGG) pathways only for miRNAs upregulated in cells infected with *L. donovani* (Tiwari et al., 2017). It was reported that miR-21 was shown to facilitate Th17 development through enhancement of the TGF-β signaling pathway by repressing SMAD-7, a negative regulator of TGF-β signaling (Murugaiyan et al., 2015). When we analyzed the TGF-β signaling protein in G_PKDL_ compared to G_THP_, we observed the important role of hsa-let7c-5p, hsa-mir21-5p, hsa-mir374-5p, hsa-mir16-5p, hsa-mir15a-5p, and hsa-mir93-5p miRNAs, which regulate these pathways. On the other hand, when we analyzed the G_PKDL_ infected host compared to G_VL_; then, we observed the significant changes in hsa-mir (155, 106, 30, 93, 6-5p) and Let-7c and 7f-5p miRNAs regulating the TGF-β signaling pathway of the host. Differential expression of miRNA (miR-135, 126, 155, 98, Let-7a-5p,7c etc.) regulates T-cell differentiation and plasticity during VL Infection (Pandey et al., 2016). It was also reported that inhibition of miR-181c-5p, miR-294-3p, miR-30e-5p, and miR-302d-3p reduces the infectivity of *Leishmania amazonensis* by modulation of Nos2, Tnf, Mcp-1/Ccl2, and RANTES/Ccl5 mRNA expression (Fernandes et al., 2019). Regulation of TGF-β by differentially expressed miRNAs may modulate host immune responses; the rapid onset and termination of diverse effector mechanisms must be efficiently controlled to prevent the adverse consequences of excessive inflammation or pathogenesis. Importantly, IL-10 and TGF-β have been associated with the activation and differentiation of Treg cells. Thus, it might be possible that the negative regulation of TGF-β from infected macrophages can activate Tregs, which then produce more TGF-β, facilitating parasite persistence. miRNAs also help in regulating autophagy by targeting BECN1, and it may have a significant impact on the treatment of leishmaniasis (Singh et al., 2016). Studies on the involvement of immunological factors have shown certain cytokines (IL-10, TGF-β, etc.) in PKDL, which may provide insight for any role in the treatment response (Verma et al., 2014). These references have clearly shown the importance of TGF-β, which is modulated by different miRNAs and validated our *in silico* data.

In addition, after *Leishmania* infection, parasites may delay the apoptosis process of the host cells, and we also observed that the mir-155, mir-335, mir-143, mir 221, mir-93, and let7c were found to be associated with negative regulation of apoptosis process, which further suggested the possibility of these miRNAs to obstruct normal functions of macrophage activation in PKDL condition. Our study also states that the upregulation of hsa-miR-146, miR-106, mir-324, mir-221, mir-9, and miR-155 have the potential to downregulate the IFN-γ signaling to help in disease progression during PKDL.

## Conclusion

Our study represents a preliminary investigation of host response modulation by host miRNAs associated with PKDL. These findings emphasize the potential of miRNAs in the regulation of macrophage effector functions and highlight their importance in leishmanial pathogenesis that can be targeted to curb PKDL. Besides, the identified miRNAs elucidate their role under immune responses in Indian PKDL patients, revealing a spectral pattern of disease progression where disease severity could be correlated inversely with proliferation and directly with TGF-beta and IL-10. This information will assist in furthering our knowledge of parasitic influences upon host cell responses in PKDL condition at a molecular level, ultimately providing opportunities to investigate new targets for rapid diagnostics or even therapeutic interventions.

## Data Availability Statement

The original contributions presented in the study are publicly available. This data can be found here: https://www.ncbi.nlm.nih.gov/, with accession number PRJNA641999.

## Author Contributions

SD, AK, and PD designed the experiments. AK, SV, MD, AS, and PD analyzed the data. AK, SD, SV, and RM wrote the manuscript. RM, SV, SD, KA, and AK performed and optimized the experiments. All authors critically revised and approved the manuscript.

## Conflict of Interest

The authors declare that the research was conducted in the absence of any commercial or financial relationships that could be construed as a potential conflict of interest.

## Footnotes

1 ftp://mirbase.org/pub/mirbase/CURRENT/genomes/hsa.gff3

2 www.mirnet.ca

## Supplementary Material

FIGURE S1 Flow chart of work done.

FIGURE S2 Scatter plot of changes in regulations of known miRNAs in G_PKDL_ versus G_VL_ **(A)** and versus G_THP_ **(B)** shows the scatter plot of upregulation of known miRNAs.

FILE S1 Fold change information of differentially expressed miRNAs.
