# Supplementary figures and images for: Differential Regulation of miRNA Profiles of Human Cells Experimentally Infected by Leishmania donovani Isolated From Indian Visceral Leishmaniasis and Post-Kala-Azar Dermal Leishmaniasis
Source: Front Microbiol. 2020 Jul 31;11:1716. doi: 10.3389/fmicb.2020.01716 (PMC7410929; doi:10.3389/fmicb.2020.01716)

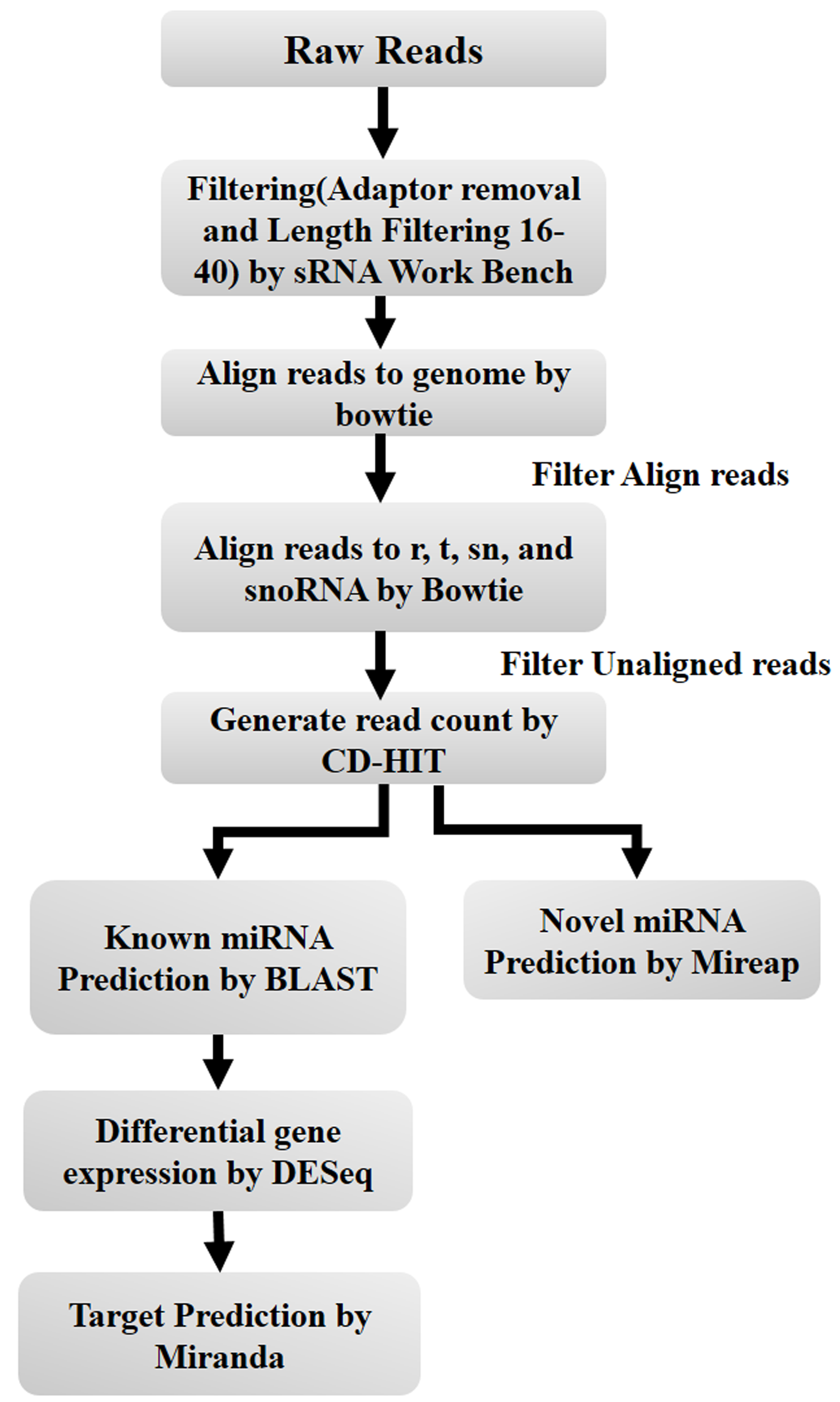

Supplement: FIGURE S1 — Flow chart of work done. [file Image_1.TIF]

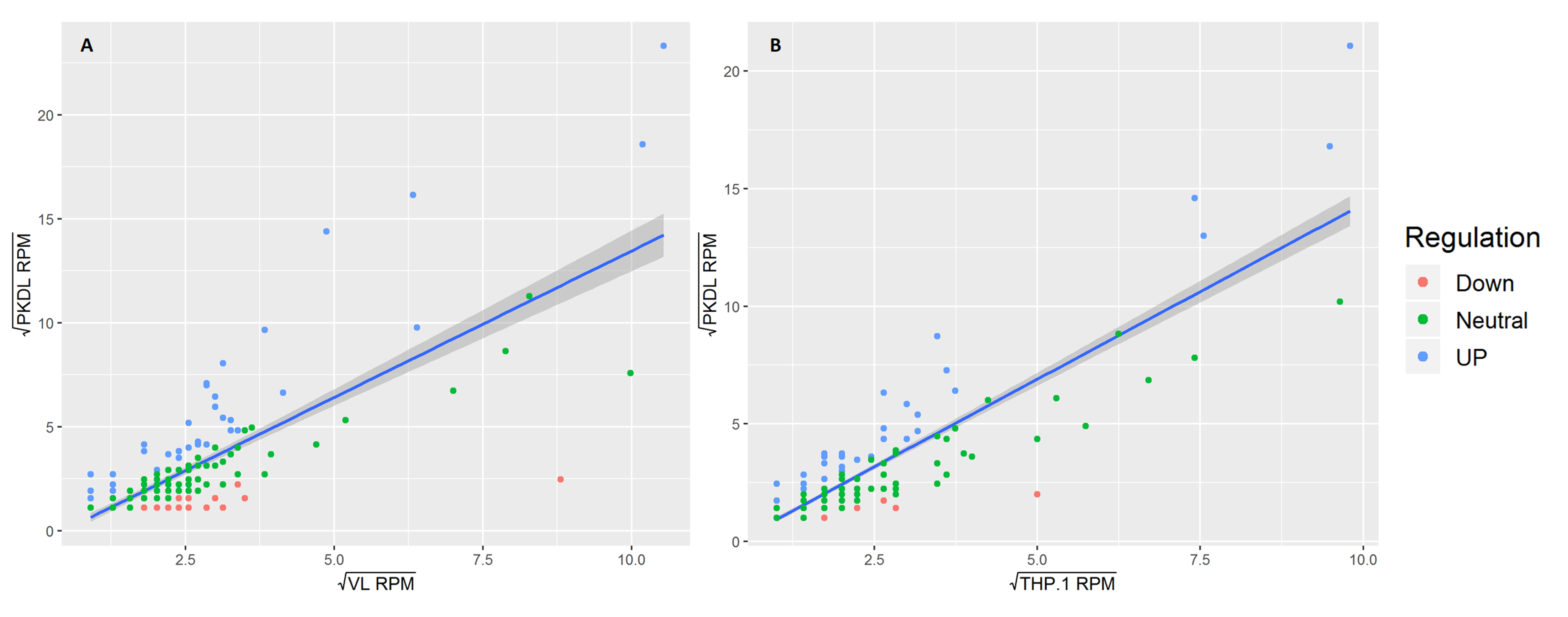

Supplement: FIGURE S2 — Scatter plot of changes in regulations of known miRNAs in GPKDL versus GVL (A) and versus GTHP (B) shows the scatter plot of upregulation of known miRNAs. [file Image_2.TIF]
